# Mortalidade Hospitalar Por Infarto do Miocárdio na América Latina e no Caribe: Revisão Sistemática e Metanálise

**DOI:** 10.36660/abc.20220194

**Published:** 2022-11-23

**Authors:** Leonardo Alves, Patrícia K. Ziegelmann, Victor Ribeiro, Carisi Polanczyk

**Affiliations:** 1 Universidade Federal do Rio Grande do Sul Programa de Pós-Graduação em Cardiologia Porto Alegre RS Brasil Programa de Pós-Graduação em Cardiologia, Universidade Federal do Rio Grande do Sul, Porto Alegre, RS – Brasil; 2 Universidade Federal do Rio Grande Faculdade de Medicina Rio Grande RS Brasil Faculdade de Medicina, Universidade Federal do Rio Grande, Rio Grande, RS – Brasil; 3 Hospital de Clínicas de Porto Alegre Porto Alegre RS Brasil Hospital de Clínicas de Porto Alegre, Porto Alegre, RS – Brasil

**Keywords:** Doenças Cardiovasculares/mortalidade, Infarto do Miocárdio/mortalidade, Pobreza/estatística e dados numéricos, América Latina, Caribe, Revisão Sistemática, Metanálise

## Abstract

**Fundamento:**

A maioria das mortes por doenças cardiovasculares ocorrem em países de renda baixa e média, e o infarto do miocárdio é uma das condições com maior risco de morte.

**Objetivos:**

Avaliar a mortalidade hospitalar por todas as causas em pacientes admitidos por infarto do miocárdio (IAMCSST e IAMSSST) na América Latina e no Caribe no ano de 2000 em diante.

**Métodos:**

Realizamos uma busca sistemática em bancos de dados eletrônicos por estudos do tipo coorte que relataram morte hospitalar por IAMCSST e IAMSSST. Foi realizada uma metanálise e um valor de p<0,05 foi considerado estatisticamente significativo.

**Resultados:**

Identificamos 38 estudos (29 com pacientes com IAMCSST, 3 com IAMSSST e 6 com IAMCSST e IAMSSST). A mortalidade por IAMCSST agrupada foi de 9,9% (IC95%: 9,1 – 10,7). Observou-se importante heterogeneidade (*I^2^* = 74% e o intervalo de predição foi de 6,6 – 14.5). A porcentagem de terapia de reperfusão e a década em que os estudos foram conduzidos explicam parte dessa heterogeneidade (*I^2^* = 54%). Quanto maior a taxa de terapia de reperfusão, menor a mortalidade hospitalar (coeficiente = −0,009, IC95%: −0,013 a −0,006, p<0,001). A mortalidade foi maior na primeira década em comparação com a mortalidade na segunda década (coeficiente = −0,14, IC95%: −0,27 a −0,02, p=0,047). A mortalidade hospitalar por IAMSSST foi de 6,3% (IC95%: 5,4 – 7,4) e a heterogeneidade foi nula.

**Conclusão:**

A mortalidade por IAMCSST em países de renda baixa e média foi maior em comparação com as taxas relatadas em outros países. Para melhorar essas estimativas, deve-se buscar um maior uso de terapia de reperfusão. A mortalidade hospitalar por IAMSSST agrupada foi similar às taxas descritas em países de alta renda. Contudo, esse dado foi baseado em poucos estudos, cuja maioria foi conduzida em dois países.

## Introdução

As doenças cardiovasculares (DCVs) são a principal causa de morte em adultos em todo o mundo. Mais de três quartos das mortes por DCVs ocorrem em países de renda baixa e média.^1^ Consequentemente, na América Latina e no Caribe, onde esses países prevalecem,^2^ as DCVs representam um peso significativo em suas economias.^3^ Na Agenda de Saúde Sustentável para as Américas 2018-2030, a Organização Panamericana de Saúde (OPAS) declarou que a redução no peso das DCVs é uma de suas metas, uma vez que essas doenças são as principais doenças não transmissíveis.^4^

A doença cardíaca isquêmica é responsável pela maioria das mortes por DCV bem como por mortes prematuras e incapacidade.^5^ Uma de suas principais manifestações é o infarto do miocárdio, uma situação comum de emergência, potencialmente fatal. O infarto do miocárdio é classificado como Infarto do Miocárdio com Elevação do Segmento ST (IAMCSST) ou como Infarto do Miocárdio sem Elevação do Segmento ST (IAMSSST), ambos com prognóstico e terapia diferentes.^6^

O manejo do infarto do miocárdio melhorou nas últimas décadas. No IAMCSST, agentes fibrinolíticos e aspirina, juntamente com intervenção coronária percutânea e agentes antiplaquetários mais potentes, diminuíram as taxas de mortalidade para 5-6%. Da mesma forma, no IAMSSST, a revascularização precoce associada com anticoagulação e novos agentes antiplaquetários também promoveram melhora dos desfechos.^7,8^

Para avaliar o manejo contemporâneo do infarto do miocárdio em países de renda baixa e média, conduzimos uma revisão sistemática para investigar mortalidade hospitalar por todas as causas em pacientes admitidos por IAMCSST e IAMSSST em hospitais na América Latina e Caribe do ano 2000 em diante.

## Métodos

Esta revisão sistemática foi conduzida de acordo com o checklist do *Meta-analysis of Observational Studies in Epidemiology* (MOOSE).^9^ O protocolo foi registrado no *International Prospective Register of Systematic Review* (PROSPERO, número CRD42019109184).

### Terminologia

Nesta revisão sistemática, a região da América Latina e do Caribe foi definida como a área geográfica composta de todos os países do continente americano, exceto os Estados Unidos, Canadá e ilhas Bermudas.^10^ Essa região tem uma população de 645 milhões; 82% moram em áreas urbanas. O Brasil e o México são os países mais populosos, contribuindo com mais da metade da população total, e Argentina, Colômbia, Peru, Venezuela e Chile com cerca de um terço da população. A região do Caribe abrange menos de 10% da população, e 70% dessa concentram-se em Cuba, Haiti, e República Dominicana.^10^ A lista de todos os países pode ser acessada no Material Suplementar.

### Critérios de seleção

Esta revisão sistemática incluiu estudos que preencheram os seguintes critérios de inclusão: (1) estudos que incluíram adultos com idade igual ou maior que 18 anos, do sexo masculino ou feminino; (2) estudos conduzidos na América Latina e no Caribe; (3) estudos que coletaram dados de pacientes admitidos em 2000 em diante; (4) estudos prospectivos e retrospectivos do tipo coorte; e (5) estudos que relataram mortalidade hospitalar por todas as causas por IAMCSST e IAMSSST.

Os critérios de exclusão consistiram em (1) estudos cujas amostras eram compostas por um grupo específico da população alvo (tais como adultos mais velhos, mulheres, diabéticas); (2) estudos cujas amostras eram compostas por um grupo com uma condição específica (como pacientes que se submeteram a uma terapia de reperfusão específica, pacientes em choque cardiogênico, pacientes que não se submeteram à terapia de reperfusão); e (3) estudos baseados em dados administrativos. Em estudos avaliando o efeito de um protocolo de tratamento comparando-se coortes antes e após sua implementação, nós selecionados o segundo período por fornecer dados mais recentes. Para coortes repetitivos, consideramos aqueles com dados originais e mais recentes. Tivemos o cuidado para evitar dupla contagem dos pacientes incluídos em diferentes coortes.

### Estratégia de busca

Conduzimos uma busca sistemática nas seguintes bases de dados eletrônicas: MEDLINE, Embase, *Web of Science*, Literatura Latino-Americana e do Caribe em Ciências da Saúde (LILACS), Centro Nacional de Informação de Ciências Médicas de Cuba (CUMED), Literatura do Caribe em Ciências da Saúde (MEDCARIB) e *Institutional Repository for Information Sharing/Pan America Health Organization* (IRIS/PAHO). A estratégia de busca combinou termos relacionados a “infarto do miocárdio” e “América Laitina e Caribe” e se restringiu a estudos publicados de 2000 em diante (Material Suplementar). Uma busca manual de referências nos artigos selecionados também foi conduzida.

Todos os artigos identificados nas diferentes fontes foram exportados para o EndNote e reunidos em um mesmo arquivo, e os artigos duplicados foram removidos.

### Seleção de artigos e extração de dados

O primeiro passo da seleção de estudos foi o rastreamento de artigos, a partir dos títulos e resumos, seguindo os critérios de elegibilidade. A segunda etapa envolveu a confirmação da elegibilidade pela leitura completa dos artigos selecionados. Nessa etapa, foram registradas as razões da exclusão do artigo e, em caso de dúvidas, os autores dos artigos foram contatados. Dois revisores independentes (L.A. e V.R.) selecionaram os estudos, e discordâncias foram resolvidas por consenso.

Extraímos características dos artigos – primeiro autor, ano de publicação, país, duração, tamanho amostral, tipo de coorte, local de recrutamento, número de centros de saúde, sistema de saúde (público/privado); características dos pacientes – características demográficas e fatores de risco (hipertensão, diabetes, tabagismo, dislipidemia); dados relacionados ao IAMCSST – classe Killip III/IV, tempo de isquemia, tipo e frequência de terapia de reperfusão – e ao IAMSSST – biomarcador de lesão miocárdica, escore de risco, terapia antitrombótica, e revascularização do miocárdio; e mortalidade hospitalar. Esse processo foi conduzido por dois revisores independentes (LA. E V.R.), e divergências foram resolvidas por consenso.

### Avaliação do risco de viés

O risco de viés nos estudos incluídos foi avaliado pela ferramenta QUIPS (*Quality in Prognosis Studies*) que contem seis domínios.^11^ Nesta revisão, usamos três desses domínios que abordam a representatividade da amostra do estudo, perda de seguimento, e a medida do desfecho. A fim de classificar a representatividade, consideramos estudos com alto risco aqueles que, no mínimo, foram conduzidos em uma única unidade de terapia intensiva ou que não realizaram (ou não informaram) recrutamento consecutivo; estudos de baixo risco aqueles com amostras populacionais; e estudos de risco moderado aqueles que não preencheram os critérios anteriores. Ainda, classificamos a perda de seguimento em baixo risco (< 10%), risco moderado (10 - 20%) ou alto risco (> 20%).

Estudos que tiveram ao menos um domínio classificado como de alto risco foram classificados como de alto risco de viés, e aqueles estudos que tiveram todos os domínios classificados como de baixo risco foram classificados como de baixo risco de viés. Estudos que não preencheram os critérios prévios foram classificados como de risco moderado de viés. Dois revisores independentes (L.A. e V.R.) conduziram essa avaliação e discordâncias foram solucionadas por consenso.

### Análise dos dados

Realizamos metanálises independentes para avaliar mortalidade hospitalar por IAMCSST e IAMSSST. A mortalidade foi exibida como proporção (número de mortes dividido pelo total de número de pacientes em risco no período de avaliação). Estimativas foram calculadas usando modelos de efeitos aleatórios (devido à heterogeneidade, esperada em estudos observacionais como o nosso), com transformação logit e método de variância inversa. Usamos o método proposto por DerSimonian e Laird para estimar a variabilidade entre estudos.

A heterogeneidade entre os estudos foi avaliada pela estatística *I*^2^,^12^ teste de Cochran e intervalo de predição de 95%. Esse intervalo fornece uma melhor perspectiva da variabilidade esperada de mortalidade entre diferentes populações consideradas nos modelos de efeito aleatórios, ou seja, a relevância clínica da heterogeneidade.^13,14^ Para identificar fontes potenciais de heterogeneidade, conduzimos análise de subgrupo (país, década do estudo) e meta-regressão. Também conduzimos análise de sensibilidade (excluindo estudos com algumas características, estudos com uma amostra pequena, estudo de alto risco de viés e estudos *outliers*) para avaliar heterogeneidade e a robustez dos resultados.

Efeitos de estudos pequenos, causados, entre outros, por viés de publicação,^15^ foram avaliados usando um gráfico de funil construído com a transformação logit de mortalidade versus o tamanho amostral. O uso do tamanho da amostra é mais preciso para avaliar estudos de proporções que uma medida de precisão.^16^ Esse efeito, observado como assimetria no gráfico de funil, foi avaliado analiticamente usando-se o teste de Peters, o qual também se baseia no tamanho da amostra.^17^ O programa R para metanálise foi usado para todas as análises.^18,19^ Um valor de p<0,05 foi considerado como estatisticamente significativo.

## Resultados

### Resultados da busca

Nossa estratégia de busca identificou 9244 artigos (01 de setembro de 2018; atualizada em 15 de abril de 2020). Após a exclusão de duplicatas, rastreamos 7597 artigos pela análise do título e do resumo; 381desses tiveram o texto completo avaliado quanto à elegibilidade. Incluímos um estudo conduzido por nosso grupo de pesquisa, que ainda não havia sido publicado até a data da última pesquisa, e cinco artigos encontrados pela busca na lista de referência de cada artigo completo incluído na revisão. Não conseguimos ter acesso a 14 artigos na íntegra mesmo após busca exaustiva. Esse processo resultou em 38 estudos: 29 sobre IAMCSST, três sobre NSTEMI e seis que avaliaram ambos (Figura Suplementar 1).

### Características do estudo

Um total de 28878 indivíduos de 35 estudos com STEMI^20–54^ e um total de 2377 indivíduos de nove estudos sobre IAMSSST^20,26,30,32,39,46,55–57^ foram incluídos na revisão. Os estudos sobre IAMCSST foram conduzidos no Brasil (n=15), Cuba (n=6), Argentina (n=5), México (n=3), Colômbia (n=2), Chile (n=1), Paraguai (n=1), Peru (n=1) e Porto Rico (n=1), enquanto estudos sobre IAMSSST foram realizados no Brasil (n=6), Argentina (n=2) e Colômbia (n=1). A maioria dos estudos foram estudos multicêntricos, prospectivos, do tipo coorte, e o departamento de emergência foi o local mais comum em que os pacientes foram recrutados. O período mediano de estudo foi de 18 meses (IQR: 12 – 37) para estudos sobre IAMCSST e 10 meses (IQR: 12 – 37) para estudos sobre IAMSSST. Características dos estudos selecionados são apresentadas nas Tabelas Suplementares 1 (IAMCSST) e 2 (IAMSSST).

Nos estudos sobre IAMCSST, a idade média variou de 55 a 65 anos de idade, e a maioria dos indivíduos eram do sexo masculino (56% ou mais em cada estudo). Em relação à seleção dos pacientes, alguns estudos usaram tempos específicos de isquemia como critérios de inclusão (até 12, 24, 36, 48 8e 72 horas). O tempo de atraso do paciente foi relatado em menos de 50% dos estudos, enquanto o tempo de atraso do sistema foi relatado em poucos estudos. A porcentagem de terapia de reperfusão variou consideravelmente entre os estudos, de 21 a 99%; cerca de 60% mostraram porcentagens abaixo de 70%. Na primeira década, a terapia de reperfusão mais frequente foi fibrinólise (estreptoquinase). A intervenção coronária percutânea foi mais frequente na segunda década; no entanto, quando fibrinólise era uma opção, escolhia -se um agente específico à fibrina. O principal motivo pela não realização de terapia e reperfusão foi o fato de os pacientes buscaram assistência 12 horas após o início dos sintomas. O atraso no sistema e o subdiagnóstico também foram mencionados.

Em estudos de IAMSSST, a idade média variou de 63 a 64 anos e a maioria dos indivíduos era do sexo masculino (60% ou mais em cada estudo). Nenhum estudo apresentou escores de risco ou relatou o uso de troponina como biomarcador de lesão miocárdica. Cinco estudos apresentaram informações sobre terapia antiplaquetária dupla e terapia de anticoagulação, e somente dois estudos mostraram dados de revascularização coronariana precoce.

### Risco de viés

O risco total de viés em estudos de IAMCSST foi de 14%, 49% e 37% para estudos de risco baixo, moderado e alto, respectivamente, e de 22%, 56% e 22% para estudos de IAMSSST com risco baixo, moderado e alto, respectivamente (Tabela Suplementar 3). O viés de seleção (domínio da representatividade) foi a principal preocupação, ao passo que a medida do desfecho e a perda de seguimento não representaram riscos.

### Desfechos de IAMCSST

**Figura 1:**
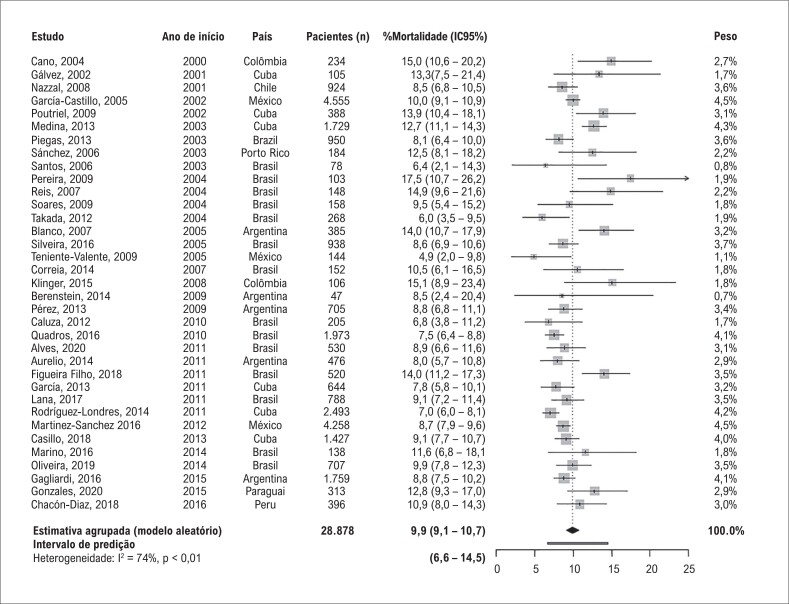
Mortalidade hospitalar agrupada em pacientes admitidos por Infarto do Miocírdio com Elevação do Segmento ST (IAMCSST) na América Latina e no Caribe do ano 2000 em diante

As taxas de mortalidade variaram substancialmente entre os estudos, de 4,9% a 17.5%. A mortalidade hospitalar agrupada foi 9,9% (IC95%: 9,1 – 10,7) (Figura 1). A amplitude do intervalo de predição (6,6 – 14,5) mostrou uma heterogeneidade relativamente importante entre os estudos. A porcentagem de variância não explicada por erro de amostragem (estatística *I*^2^) foi 74% (p<0,001). A metarregressão univaridada revelou que quanto maior a porcentagem de terapia de reperfusão, menor a mortalidade hospitalar (coeficiente −0,010, IC95%: −0,014 a −0,006, p<0,001; *I*^2^ residual = 56%) (Tabela Suplementar 4 e Figura Suplementar 2). O efeito linear sobre a taxa de mortalidade encontra-se na escala logit; assim, para uma melhor interpretação dos resultados, são apresentadas estimativas de mortalidade para algumas porcentagens de reperfusão (Tabela 1). A análise de subgrupo também identificou mortalidade hospitalar mais baixa na segunda década (2010 a 2020) ao se comparar com a primeira década (2000 a 2009) desta revisão (9,1%, IC95%: 8,2 – 10,1 *vs* 10,7%, IC95%: 9,6 – 11,9; p=0,036) (Tabela 1 e Tabela Suplementar 4). Considerando a mortalidade por país, a taxa de mortalidade mais baixa foi no Chile (8,5, IC95%: 5,3 – 13,5), e a mais alta foi na Colômbia (15%, IC95%; 10,1 – 21,7) (Tabela 1); no entanto, não foi encontrada diferença estatística entre os países (p=0,47) (Tabela Suplementar 4).

**Tabela 1:** Estimativa de mortalidade hospitalar após análise de metarregressão univariada e multivariada

Características	Mortalidade % (IC95%)	
**Análise bruta**		
**Taxa de terapia de reperfusão**		
20%	14,4 (12,3 – 16,8)	
30%	13,2 (11,6 – 15,0)	
40%	12,1 (11,0 – 13,4)	
50%	11,1 (10,3 – 12,0)	
60%	10,2 (9,5 – 10,8)	
70%	9,3 (8,7 – 9,9)	
80%	8,5 (7,8 – 9,2)	
**Década**		
Primeira	10,7 (9,6 – 11,9)	
Segunda	9,1 (8,2 – 10,1)	
**País (N de estudos)**		
Chile (1)	8,5 (5,3 – 13,5)	
México (3)	8,6 (6,5 – 11,4)	
Argentina (5)	9,6 (7,6 – 12,1)	
Brasil (15)	9,6 (8,3 – 11,0)	
Cuba (6)	10,0 (8,2 – 12,1)	
Peru (1)	10,9 (6,5 – 17,5)	
Porto Rico (1)	12,5 (7,0 – 21,2)	
Paraguai (1)	12,8 (7,7 – 20,5)	
Colômbia (2)	15,0 (10,1 – 21,7)	
**Análise ajustada**		
	**Primeira década**	**Segunda década**
**Taxa de terapia de reperfusão**		
20%	15,0 (12,8 – 17,5)	13,3 (11,2 – 15,8)
30%	13,8 (12,1 – 15,7)	12,3 (10,6 – 14,2)
40%	12,7 (11,4 – 14,2)	11,3 (10,0 – 12,7)
50%	11,7 (10,7 – 12,9)	10,4 (9,4 – 11,5)
60%	10,8 (9,9 – 11,8)	9,5 (8,8 – 10,4)
70%	9,9 (9,4 – 10,9)	8,8 (8,0 – 9,5)
80%	9,1 (8,2 – 10,2)	8,0 (7,3 – 8,9)

IC: intervalo de confiança.

No modelo de meta-regressão múltipla, somente a taxa de reperfusão e a década mantiveram associação independente com mortalidade hospitalar (Tabela Suplementar 4). Independentemente da década, o logit da mortalidade diminuiu linearmente com o aumento da taxa de reperfusão (coeficiente −0,009, IC 95%: −0,013 a −0,006, p<0,001). Independentemente da taxa de reperfusão, o logit da mortalidade foi maior na primeira década em comparação à segunda (coeficiente −0,14, IC95%: −0,27 a −0,02, p=0,047). As estimativas de mortalidade variaram de 15% a 9,1% na primeira década e de 13,3% a 8% na segunda década, dependendo da taxa de reperfusão (Tabela 1). A diferença na mortalidade ao longo das décadas variou de 1,7 ponto percentual para taxa de reperfusão de 20% a 1,1 ponto percentual para taxa de reperfusão de 80% (Tabela 1). Finalmente, a heterogeneidade diminuiu, e foi em parte explicada por essas características (*I*^2^ residual =54%).

As análises de sensibilidade excluíram estudos retrospectivos do tipo coorte, estudos com uma pequena amostra (menos de 100 participantes), estudos que usaram tempo de isquemia do paciente < 12 horas como critério de inclusão, e estudos com alto viés não afetaram muito os resultados como um todo.

### Desfechos de IAMSSST

**Figura 2:**
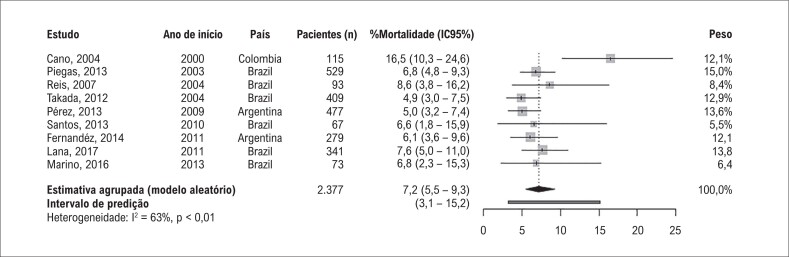
Mortalidade hospitalar agrupada em pacientes admitidos por Infarto do Miocírdio sem Elevação do Segmento ST (IAMSSST) na América Latina e no Caribe do ano 2000 em diante

As taxas de mortalidade por IAMSSST variaram de 4,9% a 8,5%, exceto um estudo cuja taxa foi de 16,5% (estudo outlier). A mortalidade hospitalar agrupada foi 7,2% (IC95%: 5,5 – 9,3) (Figura 2). A amplitude do intervalo de predição (3,2 – 15,2) mostrou uma heterogeneidade importante entre os estudos. A porcentagem de variância não explicada por erro de amostragem (estatística *I*^2^) foi de 63%. Na análise de sensibilidade (Tabela Suplementar 6), a heterogeneidade foi totalmente explicada (*I*^2^ = 0%) pela exclusão do estudo outlier (que também apresenta um viés elevado). Consequentemente, a estimativa agrupada diminuiu para 6,3% (IC95%: 5,4 – 7,4) e o intervalo de predição diminuiu para 5,1 – 7,7. A exclusão de um estudo com alto risco de viés e de três estudos com pequeno tamanho amostral (menor que 100 pacientes) não afetou os resultados. Nenhum dos estudos teve impacto individual sobre os resultados, exceto o estudo outlier como previamente mencionado.

### Efeitos de estudos pequenos

A análise visual do gráfico de funil não sugeriu efeitos de estudos pequenos sobre a mortalidade por IAMCSST, uma vez que não foi observada assimetria (Figura Suplementar 3), o que não foi corroborado pelo teste de Peters (p=0,04). Contudo, após a imputação de dois estudos hipotéticos pelo método “aparar e preencher”, ou “*trim and fill*” em inglês (análise de sensibilidade), a mortalidade agrupada não sofreu muita alteração (9,7%; IC95%: 8,9 – 10,5). Em relação aos estudos de IAMSSST, não tivemos muitos estudos para avaliar esse efeito.

## Discussão

Nesta revisão sistemática, investigamos a mortalidade hospitalar por infarto do miocárdio (IAMCSST e IAMSSST) na América Latina e no Caribe ao longo das últimas duas décadas. A mortalidade hospitalar agrupada foi de 9,3% e 6,3% para IAMCSST e IAMSSST, respectivamente, após exclusão do estudo *outlier* e do estudo com alto risco de viés. Em nosso conhecimento, esta é a primeira revisão sistemática que avaliou mortalidade por infarto do miocárdio nesta área geográfica.

A taxa de mortalidade hospitalar por IAMCSST variou entre os estudos. A principal fonte dessa heterogeneidade foi a terapia de reperfusão cuja associação com mortalidade está bem estabelecida. O mesmo fato é observado na Europa, cujos registros conduzidos por vários países mostraram taxas de mortalidade que variaram entre 4% e 13%, e grande variação também na terapia de reperfusão.^58^ Assim, o baixo uso dessa terapia, observado em muitos estudos em nossa revisão, é uma preocupação em termos da qualidade do cuidado médico. As principais razões para essa situação foram atraso do paciente em buscar cuidado médico, além do atraso no sistema e subdiagnóstico. Essas questões podem ser resolvidas principalmente com a implementação de um sistema de assistência estruturado, que envolve avaliação pré-hospitalar, triagem, e transferência, juntamente com protocolos padronizados. Essa estrutura pode melhorar o acesso a estabelecimentos de atenção terciária, diminuir o número de pacientes “elegíveis mas não tratados”, e reduzir o tempo para o tratamento.^59^ Medidas educacionais sobre dor torácica na população também devem ser implementadas. Resultados favoráveis dessas estratégias foram descritas em estudos conduzidos em países da América Latina.^22,50,60^

A mortalidade hospitalar agrupada por IAMCSST é maior que as taxas descritas em registros de países de alta renda, tais como 5,1% e 7%^61,62^ nos Estados Unidos e 6,8% no Canadá.^63^ Essa diferença pode ser devido às baixas porcentagens de terapia de reperfusão. Esse fato é corroborado pelo estudo que avaliou desfechos em pacientes com STEMI em ensaios clínicos que mostraram uma associação negativa entre mortalidade e renda nacional bruta.^64^ Essa associação foi independente de outros preditores, tais como gravidade dos casos, tempo de isquemia e manejo de perfusão.

Outra fonte de heterogeneidade encontrada nos estudos com pacientes com IAMCSST relacionou-se ao período em que os estudos foram conduzidos. Na primeira década dessa revisão, observamos uma maior mortalidade que na segunda década, o que pode ser devido ao uso predominante de agentes não específicos à fibrina para a fibrinólise e menor uso de terapia antitrombótica. Deve-se salientar que o resultado dessa fonte de heterogeneidade foi muito próximo ao limite arbitrário da significância estatística.

Finalmente, a mortalidade hospitalar variou ente os países em que os estudos foram desenvolvidos, mas essa fonte de heterogeneidade não foi estatisticamente significativa. Apesar de a América Latina e o Caribe serem compostos de países de renda baixa e média, existem diferenças em suas rendas nacionais brutas e seus sistemas de saúde.^64^ Nesse caso, o fato de a presente revisão sistemática não ter poder estatístico suficiente pode ter influenciado no resultado.

Dois grandes registros de IAMCSST conduzidos na América Latina (México e Brasil) devem ser destacados. Esses estudos relataram a mortalidade cardiovascular hospitalar^60^ e em 30 dias^65^ em vez de mortalidade hospitalar por todas as causas, como em nossa revisão.^60,65^ No registro mexicano, 71% dos pacientes receberam terapia de reperfusão e a mortalidade cardiovascular foi 9,4% (após implementação do protocolo de manejo). Essa taxa também é mais elevada que as taxas encontradas nos registros em países de alta renda. No registro brasileiro, a terapia de reperfusão foi aplicada em 88% dos pacientes enquanto a mortalidade cardiovascular em 30 dias foi de 3,4%. Essa taxa foi mais baixa que aquelas observadas em países de alta renda embora essa tenha considerado apenas mortes cardiovasculares. As razões para esse fato podem incluir a participação de centros de tratamento cardíaco de referência, além dos métodos de amostragem e recrutamento usados.

Há limitações a serem consideradas. Alguns estudos adotaram limites diferentes de tempos de isquemia devido ao atraso do paciente como critério de inclusão (outros não mencionaram se os usaram). Uma vez que o tempo de isquemia está associado com mortalidade, esses estudos poderiam selecionar pacientes com diferentes prognósticos. Ainda, a falta de dados sobre tempo de isquemia (atraso do paciente ou do sistema) nos estudos não permitiu avaliá-lo como uma fonte de heterogeneidade, uma vez que a mortalidade está associada não só com a realização de terapia de reperfusão, como também com o período em que foi realizada. Outras fontes potenciais de heterogeneidade, tais como idade e proporção de mulheres não foram avaliadas devido à falta de informação. Por fim, deve-se considerar a questão da representatividade dos estudos. Esta revisão de estudos sobre IAMCSST incluiu apenas nove países, e a maioria dos estudos foram conduzidos em serviços de saúde bem estruturados, que geralmente apresentam melhores resultados.

A mortalidade hospitalar por IAMCSST entre os estudos, após excluir o estudo outlier e de alto risco de viés. As estimativas acumuladas foram similares às taxas de mortalidade de grandes registros, tais como 5% no estudo GRACE e 7,6% no registro Kaiser.^62,66^ Contudo, há algumas ressalvas a ser consideradas nessas análises. A escassez de dados sobre mortalidade hospitalar por IAMSSST isolada deve-se ao fato de que, na maioria dos estudos, a mortalidade por IAMSSST estar combinada com outras causas como angina instável. Além disso, os estudos foram conduzidos principalmente em dois países (Brasil e Argentina), o que pode prejudicar a generalização das estimativas para toda a região. Ainda, os estudos não apresentaram nenhum escore de risco; assim, não conseguimos avaliar a gravidade da população estudada para fins comparativos.

Finalmente, o risco global de viés foi classificado em alto e moderado de acordo com o viés de seleção. Assim, deve-se prestar atenção aos métodos de amostragem para evitar uma estimativa enviesada. Ainda, a definição do domínio de representatividade nesta revisão foi arbitrária, o que foi uma limitação. Consequentemente, esses fatos devem ser considerados na avaliação das estimativas de mortalidade hospitalar.

## Conclusão

A mortalidade hospitalar por IAMCSST agrupada em países de renda baixa e média foi elevada em comparação a taxas encontradas em países de renda alta. Para melhorar essas estimativas, é fundamental aumentar a porcentagem da terapia de reperfusão, o que pode ser alcançado focando-se na organização do sistema de saúde e na saúde da população. A mortalidade hospitalar por IAMSSST foi similar às encontradas em países de alta renda; no entanto, tal estimativa baseou-se em poucos estudos, cuja maioria foi conduzida em dois países. Assim, em relação aos dados de IAMSSST, devem-se avaliar mais registros de diferentes países para se obter uma estimativa mais precisa. Por fim, pesquisadores devem focar na qualidade dos métodos de amostragem e de recrutamento para se evitar o risco de viés e, consequentemente, melhorar as estimativas.

## *Material suplementar

Para informação adicional, por favor, clique aqui.
