# Supplementary material for: Mortalidade Hospitalar Por Infarto do Miocárdio na América Latina e no Caribe: Revisão Sistemática e Metanálise
Source: Arq Bras Cardiol. 2022 Nov 23;119(6):970–8. [Article in Portuguese] doi: 10.36660/abc.20220194 (PMC9814809; doi:10.36660/abc.20220194)
Supplement: Translation

# Translation: Hospital Mortality from Myocardial Infarction in Latin America and the Caribbean: Systematic Review and Meta-Analysis

**Authors:** Leonardo Alves, Patrícia K. Ziegelmann, Victor Ribeiro, Carisi Polanczyk

**Affiliations:** 1 Universidade Federal do Rio Grande do Sul Programa de Pós-Graduação em Cardiologia Porto Alegre RS Brazil Programa de Pós-Graduação em Cardiologia, Universidade Federal do Rio Grande do Sul, Porto Alegre, RS – Brazil; 2 Universidade Federal do Rio Grande Faculdade de Medicina Rio Grande RS Brazil Faculdade de Medicina, Universidade Federal do Rio Grande, Rio Grande, RS – Brazil; 3 Hospital de Clínicas de Porto Alegre Porto Alegre RS Brazil Hospital de Clínicas de Porto Alegre, Porto Alegre, RS – Brazil

**Keywords:** Cardiovascular Diseases/mortality, Myocardial Infarction/mortality, Poverty/statistics & Numeral data, Latin America, Caribbean Region, Systematic Review, Meta-Analysis

## Abstract

**Background:**

Most cardiovascular deaths occur in low- and middle-income countries and myocardial infarction is one of the main life-threatening conditions.

**Objective:**

We assessed all-cause in-hospital mortality in patients admitted for myocardial infarction (STEMI and NSTEMI) in Latin America and the Caribbean from 2000 onward.

**Methods:**

We systematically searched in electronic bibliographic databases for cohort studies which reported in-hospital mortality due to STEMI and NSTEMI. A meta-analysis was performed and a p-value < 0.05 was considered significant.

**Results:**

We identified 38 studies (29 STEMI, 3 NSTEMI and 6 both). Pooled STEMI in-hospital mortality was 9.9% (95% CI: 9.1 – 10.7). Heterogeneity was not trivial (I^2^ = 74% and prediction interval = 6.6 – 14.5). The percentage of reperfusion therapy and decade explain part of the heterogeneity (I^2^ = 54%). The higher the rate of reperfusion therapy, the lower the in-hospital mortality (coefficient = −0.009, 95% CI: −0.013 to −0.006, p<0.001). This mortality was higher in the first decade as compared with the second (coefficient = −0.14, 95% CI: −0.27 to −0.02, p=0.047). Pooled NSTEMI in-hospital mortality was 6.3% (95% CI: 5.4 – 7.4) and heterogeneity was null.

**Conclusion:**

Pooled STEMI in-hospital mortality in low- and middle-income countries was high in comparison with rates reported in high income countries. To improve these estimates, higher use of reperfusion therapy must be pursued. Pooled NSTEMI in-hospital mortality was similar to the ones found in high-income countries; however, it was based on few studies and most of them were carried out in two countries.

## Introduction

Cardiovascular diseases (CVDs) are the main cause of mortality among adults worldwide. Over three quarters of CVD deaths occur in low- and middle-income countries.^1^ As a result, in Latin American and the Caribbean, where these countries prevail,^2^ CVD represent a significant burden on their economies.^3^ In the Sustainable Health Agenda for the Americas 2018-2030, the Pan American Health Organization (PAHO) declared that decrease in the CVD burden is one of its goals since these disorders are the main noncommunicable diseases.^4^

Ischemic heart disease is responsible for most deaths caused by CVD as well as for premature death and disability.^5^ One of its main clinical manifestations is myocardial infarction, a common life-threatening emergency. It is classified as ST-segment elevation myocardial infarction (STEMI) or non-ST-elevation myocardial infarction (NSTEMI), and both have different prognosis and therapy.^6^

Management of myocardial infarction has improved in last decades. In STEMI, fibrinolytic agents and aspirin, along with percutaneous coronary intervention and more powerful new antiplatelet agents, have decreased hospital mortality rates to 5-6%. Likewise, in NSTEMI, early revascularization associated with anticoagulation and new antiplatelet agents has also improved the outcomes.^7,8^

In order to evaluate the contemporary management of myocardial infarction in low- and middle-income countries, we carried out a systematic review to assess all-cause in-hospital mortality in patients admitted for STEMI and NSTEMI in hospitals in Latin America and the Caribbean from 2000 onward.

## Methods

This systematic review was performed according to the Meta-analysis of Observational Studies in Epidemiology (MOOSE) checklist.^9^ The protocol was registered in the International Prospective Register of Systematic Reviews (PROSPERO, number CRD42019109184).

### Terminology

In this systematic review, the Latin America and the Caribbean region was defined as the geographic composed of all countries in the American continent, except the USA, Canada and the Bermuda Islands.^10^ The region has a population of 645 million; 82% live in urban areas. Brazil and Mexico are the most populous countries, accounting for more than a half of the total population, and Argentina, Colombia, Peru, Venezuela and Chile for about one third. The Caribbean region accounts for less than 10% of the population and approximately 70% of this concentrates in Cuba, Haiti and Dominican Republic.^10^ The list of all countries may be accessed in the Supplementary Material.

### Selection criteria

This systematic review included studies that met the following inclusion criteria: (1) included male and female adults who are 18 years old and older; (2) carried out in countries in Latin America and the Caribbean; (3) collected data from patients admitted from 2000 onward; (4) prospective or retrospective cohort studies; and (5) reported all-cause in-hospital mortality due to STEMI and/or NSTEMI.

Exclusion criteria consisted of studies (1) whose samples were a specific group of the target population (such as older adults, women, diabetics); (2) whose samples were a group with a specific condition (such as patients who underwent a specific reperfusion therapy, who were in cardiogenic shock, who did not undergo reperfusion therapy); and (3) studies based on administrative data. In studies using before-after cohorts to evaluate the effect of implementing a management protocol, we selected the second period, as it would provide more recent data. For repetitive cohorts, we considered the ones with original and more recent data. We were careful to avoid double counting of patients included in different cohorts.

### Search strategy

A systematic search was carried out in the following electronic databases: MEDLINE, Embase, Web of Science, Latin America and Caribbean Health Science Literature (LILACS), National Center of Cuba Medical Information (CUMED), Caribbean Health Sciences Literature (MEDCARIB) and Institutional Repository for Information Sharing/Pan America Health Organization (IRIS/PAHO). The search strategy combined terms related to “myocardial infarction” and “Latin America and the Caribbean” and was restricted to studies published from 2000 onward (Supplementary Material), and was not limited by language. A manual search of the references of selected articles was also conducted.

All reports identified in the different sources were exported to EndNote, gathered in a same file, and duplicates were removed.

### Study selection and data extraction

The first step of study selection comprised the screening of reports, in agreement with eligibility criteria, through reading titles and abstracts. The second step involved the confirmation of eligibility through reading the full texts of the selected studies. In this step, reasons for exclusion were registered and, if there was any doubt, the authors were contacted. Two independent reviewers (L.A. and V.R.) selected the studies, and disagreements were resolved by consensus.

We extracted study characteristics (first author, year of publication, country, time period, sample size, type of cohort, local of recruitment, number of health centers, funding health system); patient characteristics (demographic characteristics and risk factors – hypertension, diabetes, smoking and dyslipidemia); STEMI-related data (III/IV Killip classes, ischemic time and reperfusion therapy percentage and type) and to NSTEMI studies (biomarker of myocardial injury, risk score, antithrombotic therapy and myocardial revascularization); and in-hospital mortality. This process was conducted by two reviewers independently (L.A. and V.R.) and disagreements were resolved by consensus.

### Risk of bias assessment

The overall risk of bias in included studies was assessed by the Quality in Prognosis Studies (QUIPS) tool which consists of six domains.^11^ In this review, we used three of them that address representativeness of the study sample, loss to follow-up, and the outcome measurement. In order to rate representativeness, we considered high-risk studies those that, at least, conducted in a single intensive care unit or did not perform consecutive recruitment (or not reported); low-risk studies those with population-based samples; and moderate-risk studies those that did not meet the previous criteria. We rated loss to follow-up as low risk (< 10%), moderate risk (10 – 20%) or high risk (> 20%).

Studies that had at least one domain rated as high risk were classified into overall high risk of bias, while the ones that had all domains rated as low risk were classified into overall low risk of bias. Studies that did not meet the previous criteria were classified into overall moderate risk of bias. Two independent reviewers (L.A. and V.R.) conducted this evaluation and disagreement was solved by consensus.

### Data analysis

We performed independent meta-analyses to assess STEMI and NSTEMI in-hospital mortality. Mortality was exhibited as proportion (number of deaths divided by the total number of patients at risk in the period under evaluation). Pooled estimates were calculated by using the random effect models (due to heterogeneity, which is expected in observational studies like ours) with logit transformation and inverse variance method (as a sensitive analysis, GLM was adjusted and the difference in results was unnoticeable). We used the DerSimonian and Laird method to estimate the between-study variability.

Heterogeneity across studies was evaluated by *I*^2^ statistics,^12^ Cochran test and 95% prediction interval. This interval gives a better picture of the mortality variability expected among different populations considered in the random effect models, that is, the clinical relevance of heterogeneity.^13,14^ To identify potential sources of heterogeneity, we conducted subgroup analysis (country, decade of the study) and meta-regression. We also conducted sensitivity analysis (excluding studies with some characteristics, studies with a small sample size, high bias risk studies and outlier studies) to evaluate heterogeneity and the robustness of results.

Small-study effects, which has publication bias as one of the causes,^15^ were evaluated by funnel plot that was constructed with the logit transformation of mortality against the sample size. The use of sample size is more accurate to evaluate proportion studies than the use of a measure of precision.^16^ This effect, which is observed by asymmetry on funnel plot, was evaluated analytically by the Peters test that is also based on sample size.^17^ R software meta package was used to perform all analyses.^18,19^ A P value below < 0.05 was considered statistically significant.

## Results

### Search results

Our search strategy identified 9,244 reports (1st September 2018; updated on 15th April 2020). After the exclusion of duplicates, we screened 7,597 reports through title and abstract analysis of which 381 full texts were assessed for eligibility. We included one study carried out by our research group that had not been published up to the date of the search update and five reports found by screening the reference list of each full text included in the review. We could not get access to 14 full text articles despite exhaustive search. This process resulted in 38 studies: 29 on STEMI, three on NSTEMI and six that evaluated both (Supplementary Figure 1).

**Figure 1:** Pooled in-hospital mortality in patients admitted due to STEMI in Latin America and the Caribbean from 2000 onward.

### Study characteristics

A total of 28,878 individuals from 35 STEMI studies^20–54^ and a total of 2,377 individuals from nine NSTEMI studies^20,26,30,32,39,46,55–57^ were included in this review. STEMI studies were conducted in Brazil (n=15), Cuba (n=6), Argentina (n=5), Mexico (n=3), Colombia (n=2), Chile (n=1), Paraguay (n=1), Peru (n=1) and Puerto Rico (n=1), while NSTEMI ones were conducted in Brazil (n=6), Argentina (n=2) and Colombia (n=1). Most studies were multicenter prospective cohort studies and emergency rooms were the most frequent locals of recruitment. Median study period was 18 months (IQR: 12 – 37 months) for STEMI studies and 10 months (IQR: 12 – 37 months) for NSTEMI studies. Characteristics of the selected studies are shown in Supplementary Table 1 (STEMI) and Supplementary Table 2 (NSTEMI).

**Table 1:** In-hospital mortality estimation following univariate and multivariable meta-regression analysis

Characteristic	Mortality % (95% CI)	
**Crude Analysis**		
**Reperfusion therapy rate**		
20%	14.4 (12.3 – 16.8)	
30%	13.2 (11.6 – 15.0)	
40%	12.1 (11.0 – 13.4)	
50%	11.1 (10.3 – 12.0)	
60%	10.2 (9.5 – 10.8)	
70%	9.3 (8.7 – 9.9)	
80%	8.5 (7.8 – 9.2)	
**Decade**		
First	10.7 (9.6 – 11.9)	
Second	9.1 (8.2 – 10.1)	
**Country (N of studies)**		
Chile (1)	8.5 (5.3 – 13.5)	
Mexico (3)	8.6 (6.5 – 11.4)	
Argentina (5)	9.6 (7.6 – 12.1)	
Brazil (15)	9.6 (8.3 – 11.0)	
Cuba (6)	10.0 (8.2 – 12.1)	
Peru (1)	10.9 (6.5 – 17.5)	
Puerto Rico (1)	12.5 (7.0 – 21.2)	
Paraguay (1)	12.8 (7.7 – 20.5)	
Colombia (2)	15.0 (10.1 – 21.7)	
**Adjusted Analysis**		
	**First decade**	**Second decade**
**Reperfusion therapy rate**		
20%	15.0 (12.8 – 17.5)	13.3 (11.2 – 15.8)
30%	13.8 (12.1 – 15.7)	12.3 (10.6 – 14.2)
40%	12.7 (11.4 – 14.2)	11.3 (10.0 – 12.7)
50%	11.7 (10.7 – 12.9)	10.4 (9.4 – 11.5)
60%	10.8 (9.9 – 11.8)	9.5 (8.8 – 10.4)
70%	9.9 (9.4 – 10.9)	8.8 (8.0 – 9.5)
80%	9.1 (8.2 – 10.2)	8.0 (7.3 – 8.9)

CI: confidence interval.

In STEMI studies, mean age varied from 55 to 65 years old and most individuals were males (56% or more in each study). Regarding patient selection, some studies used specific ischemic times as inclusion criterion (up to 12, 24, 36, 48 and 72 hours). Patient delay time was reported in less than 50% of the studies while system delay time was reported in very few studies. The percentage of reperfusion therapy varied considerably across studies, from 21% to 99%; around 60% of them showed percentages below 70%. In the first decade, the most frequent reperfusion therapy was fibrinolysis (streptokinase). Primary percutaneous coronary intervention was more frequent in the second decade; however, when fibrinolysis was an option, a fibrin-specific agent was chosen. The main cause of no reperfusion therapy was the fact that patients looked for assistance 12 hours after symptom onset. System delay time and under-diagnosis were also mentioned.

In NSTEMI studies, mean age varied from 63 to 65 years old and most individuals were males (60% or more in each study). No study presented any risk scores or reported exclusive use of troponin as biomarker of myocardial injury. Five studies reported information about dual antiplatelet and anticoagulation therapy and only two reported data on early coronary revascularization.

### Risk of bias

Overall risk of bias in STEMI studies was 14%, 49% and 37% for low, moderate and high-risk studies, respectively, and 22%, 56% and 22% for low, moderate and high-risk studies on STEMI, respectively (Supplementary Table 3). The selection bias (representativeness domain) was the primary concern while outcome measurement and loss to follow-up did not represent any risk.

### STEMI outcomes

Mortality rates varied substantially across the studies, from 4.9% to 17.5%. Pooled in-hospital mortality was 9.9% (95% CI: 9.1 – 10.7) (Figure 1). Width of the prediction interval (6.6 – 14.5) showed non-trivial heterogeneity across studies. Percentage of variance not explained by sampling error (*I*^2^ statistics) was 74% (p <0.001). Univariate meta-regression revealed that the higher the percentage of reperfusion therapy, the lower the in-hospital mortality (coefficient −0.010, 95% CI: −0.014 to −0.006, p<0.001; residual *I*^2^ = 56%) (Supplementary Table 4 and Supplementary Figure 2). The linear effect on mortality rate is on the logit scale; thus, to improve the interpretation of results, mortality estimates for some reperfusion percentages are shown (Table 1). Subgroup analysis also identified lower in-hospital mortality in the second decade (2010 to 2020) by comparison with the first decade (2000 to 2009) of this review (9.1%, 95% CI: 8.2 – 10.1 *vs* 10.7%, 95% CI: 9.6 – 11.9; p=0.036) (Table 1 and Supplementary Table 4). Considering mortality by country, the lowest in-hospital mortality was in Chile (8.5, 95% CI: 5.3 – 13.5) while the highest was in Colombia (15%, 95% CI; 10.1 – 21.7) (Table 1); however, no statistical difference was found among counties (p=0.47) (Supplementary Table 4).

**Figure 2:** Pooled in-hospital mortality in patients admitted due to NSTEMI in Latin America and the Caribbean from 2000 onward.

In the multiple meta-regression model, only reperfusion rate and decade kept independently associated with in-hospital mortality (Supplementary Table 4). Regardless of the decade, logit of mortality decreased linearly when reperfusion rate increased (coefficient −0.009, 95% CI: −0.013 to −0.006, p<0.001). Regardless of the reperfusion rate, logit of mortality was higher in the first decade by comparison with the second one (coefficient −0.14, 95% CI: −0.27 to −0.02, p=0.047). Mortality estimates varied from 15% to 9.1% in the first decade and from 13.3% to 8% in the second decade, depending on reperfusion rate (Table 1). Difference in mortality throughout decades varied from 1.7 percentage point for 20% reperfusion rate to 1.1 percentage point for 80% rate (Table 1). Finally, heterogeneity decreased and was partially explained by these characteristics (residual *I*^2^=54%).

Sensitivity analyses excluding retrospective cohort studies, studies with a small sample size (below 100 patients), studies which used patient delay ischemic time < 12 h as inclusion criterion and high bias studies did not affect much overall results (Supplementary Table 5). None of the studies individually impacted results.

### NSTEMI outcomes

NSTEMI mortality ranged from 4.9% to 8.6% across the studies, except one study whose rate was 16.5% (outlier study). Pooled NSTEMI in-hospital mortality was 7.2% (95% CI: 5.5 – 9.3) (Figure 2). The width of prediction interval (3.2 – 15.2) showed a substantial heterogeneity across studies. Percentage of variance not explained by sampling error (*I*^2^ statistics) was 63%. In sensitivity analysis (Supplementary Table 6), heterogeneity was totally explained (*I*^2^=0%) by exclusion of the outlier study (which is also a high bias one). As a result, the pooled estimate decreased to 6.3% (95% CI: 5.4 – 7.4) and the prediction interval narrowed to 5.1 – 7.7. Exclusion of one study with high bias risk and of three studies with a small sample size (below 100 patients) did not affect results. None of the studies individually impacted results, except the outlier study as previously mentioned.

#### Small-study effects

Visual inspection of funnel plot did not suggest small-study effects on STEMI mortality since asymmetry was not observed (Supplementary Figure 3), but it was not supported by the Peters test (p = 0.04). However, after the imputation of two hypothetical studies by the trim-and-fill method (sensitivity analysis), pooled mortality did not change much (9.7%; 95% CI: 8.9 – 10.5). Regarding NSTEMI studies, we did not have enough studies to assess this effect.

## Discussion

In this systematic review, we investigated in-hospital mortality due to myocardial infarction (STEMI and NSTEMI) in Latin America and the Caribbean over the two last decades. Pooled in-hospital mortality was 9.9% and 6.3% for STEMI and NSTEMI, respectively, after exclusion of the outlier study with high-bias risk. To the best of our knowledge, it is the first systematic review that evaluated mortality due to myocardial infarction in this geographical area.

In-hospital mortality rate for STEMI varied among studies. The main source of this heterogeneity was the reperfusion therapy whose association with mortality has been well-established. The same fact is observed in Europe, where registries carried out by several countries showed mortality rates that ranged from 4% to 13% while reperfusion therapy also varied much.^58^ Therefore, low use of this therapy, which was observed in many studies in our review, is a concern in terms of the quality of medical care. The main reasons for this situation were patient delay in seeking medical care, besides system delay and under-diagnosis. These issues can be solved mainly with implementation of well-structured system of care which involves prehospital evaluation, triage, and transfer together with standardized protocols. This structure can improve access to tertiary care facilities, decrease the number of “eligible, but untreated” patients and shorten time-to-treatment.^59^ Educational measures about chest pain in the population must also implemented. Favorable results of these strategies were described by studies conducted in Latin American countries.^22,50,60^

Pooled in-hospital mortality rate for STEMI is higher than the ones found in registries in high-income countries, such as 5.1% and 7%^61,62^ in the United States and 6.8% in Canada.^63^ This difference may be due to low perfusion therapy percentages. This fact is also supported by the study that evaluated outcomes in STEMI patients in clinical trials which found negative association between mortality and gross national income.^64^ This association was independent of other predictors, such as severity of cases, ischemic time and perfusion management.

Another source of heterogeneity that we found in STEMI studies was related to the period in which studies were carried out. In the first decade of this review, we observed higher mortality than in the second one, which may be due to predominant use of non-fibrin-specific agents for fibrinolysis and less anti-thrombotic therapy. It should be highlighted that the result of this source of heterogeneity was very close to the arbitrary limit of statistical significance.

Finally, in-hospital mortality varied among the countries where the studies were carried out, but this source of heterogeneity was not statistically significant. Although the Latin America and the Caribbean are composed of low- and middle-income countries, there are differences in their gross national incomes and health systems.^64^ In this case, the fact that this systematic review did not have enough statistical power any power may have influenced the result.

Two large STEMI registries conducted in Latin America (Mexico and Brazil) should also be highlighted. They reported in-hospital^60^ and 30-day cardiovascular mortality^65^ rather than all-cause in-hospital mortality, as in our review.^60,65^ In the Mexican registry, 71% of patients received reperfusion therapy and cardiovascular mortality was 9.4% (after implementation of management protocol). This rate is also higher than the ones found in registries in high income countries. In the Brazilian registry, reperfusion therapy was used in 88% of patients while 30-day cardiovascular mortality was 3.4%. This rate was lower than the ones observed in high income countries although it considered only cardiovascular deaths. Reasons for this fact may include the participation of referral cardiac care centers, besides sampling and recruitment methods under use.

There are limitations to be considered. Some studies used different limits of ischemic times due to patient delay as an inclusion criterion (others did not mention whether they used it). Since ischemia time is associated with mortality, these studies could select patients with different prognosis. Likewise, lack of data on ischemia time (patient delay and system delay) in studies did not allow to evaluate it as a source of heterogeneity since mortality is not only associated with performing reperfusion therapy, but also with the time period in which it is performed. Other potential sources of heterogeneity, such as mean age and proportion of females were not also evaluated due to lack of information. Finally, concern about representativeness of studies should be considered. This systematic review of STEMI studies included only nine countries, and most studies were conducted in well-structured health services which usually have better results.

In-hospital mortality for NSTEMI across studies did not change after excluding the one outlier study, with a high bias risk. Pooled estimates were similar to the mortality rates of large registries, such as 5% in the GRACE study and 7.6% in the Kaiser registry.^62,66^ However, there are caveats to be considered in these analyses. The shortage of data on in-hospital mortality from NSTEMI alone is due to the fact that most studies have combined mortality from NSTEMI with from other conditions like unstable angina. In addition, the studies were carried out mainly in two countries (Brazil and Argentina), which can harm generalization of the estimate in the region. The studies did not report any risk score; therefore, we could not evaluate and compare the severity level of the population under study.

Finally, the overall risk of bias was classified into high and moderate risk according to the selection bias. Therefore, attention must be paid to sampling methods in order to avoid biased estimate. In addition, definition of the representativeness domain in this review was arbitrary, which was a limitation. As a result, these facts should be taken into account when in-hospital mortality estimates are considered.

## Conclusion

Pooled STEMI in-hospital mortality in low- middle-income countries was high in comparison with rates found in high income countries. To improve these estimates, it is fundamental to increase the percentage of reperfusion therapy, which can be reached by focusing on organization of the health care system and population education. Pooled NSTEMI in-hospital mortality was similar to the ones found in high-income countries; however, it was based on few studies and most of them were carried out in two countries. Therefore, regarding NSTEMI data, more registries from different countries must be addressed to obtain a more accurate estimate. Finally, researchers must focus on quality of both sampling and recruitment methods in order to avoid bias risk and, consequently, improve estimates.

## *Supplemental Materials

For additional information, please click here.
